# The role of facial cues in signalling cooperativeness is limited and nuanced

**DOI:** 10.1038/s41598-024-71685-9

**Published:** 2024-09-24

**Authors:** Johannes Lohse, Santiago Sanchez-Pages, Enrique Turiegano

**Affiliations:** 1 Institute for Economics, Leuphana University Lüneburg, Lüneburg, Germany; 2 Department of Economics, University of Birmingham, Birmingham, UK; 3 Department of Political Economy, King’s College London, London, UK; 4 Department of Biology, Universidad Autonoma de Madrid, Madrid, Spain

**Keywords:** Cooperation, Facial images, Predictability, Signaling, Human behaviour, Social evolution, Social anthropology

## Abstract

Humans display a remarkable tendency to cooperate with strangers; however, identifying prospective cooperation partners accurately before entering any new relationship is essential to mitigate the risk of being exploited. Visual appearance, as inferrable, for example, from facial images on job portals and dating sites, may serve as a potential signal of cooperativeness. This experimental study examines whether static images enable the correct detection of an individual’s propensity to cooperate. Participants first played the Prisoner’s Dilemma (PD) game, a standard cooperation task. Subsequently, they were asked to predict the cooperativeness of participants from a prior PD study relying solely on their static facial photographs. While our main results indicate only marginal accuracy improvements over random guessing, a more detailed analysis reveals that participants were more successful at identifying cooperative tendencies similar to their own. Despite no detectable main effect in our primary treatment variations (time pressure versus time delay), participants exhibited increased accuracy in identifying male cooperators under time pressure. These findings point towards a limited yet nuanced role of static facial images in predicting cooperativeness, advancing our understanding of non-behavioral cues in cooperative interactions.

## Introduction

Humans constantly interact with each other. The outcomes of these interactions often hinge on decisions that individuals make independently. This is the case, for instance, in cooperative interactions where one person voluntarily incurs a cost to generate a joint benefit shared with a counterpart. However, such cooperative acts are susceptible to exploitation by opportunistic counterparts who free-ride on the efforts of others. This potential for exploitation may deter future opportunities for mutually beneficial cooperation and hence result in substantial losses in social welfare^1–3^. Thus, the ability to correctly predict others’ cooperative inclinations is advantageous in establishing new relationships and sustaining cooperative interactions. Signals of cooperativeness typically emerge from observing past behavior in repeated interactions or from receiving indirect information on the reputation built in interactions with others, as exemplified by ratings on websites or reference letters^4–6^. However, in the absence of information on past behaviour, the visual appearance of a potential counterpart could serve as an alternative cue.

The ability to correctly predict others’ cooperative behaviors holds such an advantage that it might result from an evolutionary mechanism^7,8^. This mechanism prompts evolutionary changes in assessing a person’s likelihood to cooperate based on various observable characteristics^9–11^. As a result, opportunists gain from pretending to harbor cooperative intentions by imitating any easily observable characteristic that identifies probable cooperators. This, in turn, encourages the emergence of increasingly complex and difficult-to-imitate signals of cooperativeness and the development of more sophisticated detection abilities^10^. As a consequence, it is commonly assumed that any observable indication of non-cooperative intent must be subtle and changing; if the differences between cooperators and opportunists were instead obvious, opportunists would never be successful in exploiting cooperators and would disappear from the population over time. In line with this proposition, existing studies on this evolutionary cat-and-mouse game find only a moderate ability to detect cooperators within a population and the emergence of remarkably subtle indicators of cooperative intent^12^.

A better-than-chance ability to predict whether a person will cooperate has been observed when experimental subjects made their choices after a brief face-to-face encounter^13–16^. Detection abilities also appear to be significantly better than chance in experiments using silent video clips of partners^12,17–20^. Non-verbal cues thus seem to help detect cooperators. In particular, individuals’ micro-expressions during decision-making appear to play a role in these non-verbal cues, as indicated by higher detection rates in static photos taken precisely at the moment the decision is made^9,21,22^, although the evidence is mixed^12^. The ability to detect cooperators from visual appearance, therefore, seems to be better than chance only when involuntary movements and gestures can be observed^17^, but not from observing static images alone^23^. Only a small set of studies have documented better-than-chance prediction abilities using static images not captured concurrently with participants’ choices. In these studies, certain observable attributes of the individuals in the photos, such as their gender or facial dimorphism^24^, were observed to affect detection ability. When it comes to trustworthiness - a behavior closely linked to cooperation - the evidence is equally mixed. Some studies have found that individuals can judge people’s trustworthiness from static images^25^, while other studies suggest that accuracy is no better than chance^23,26^.

And yet, in our daily lives, decisions about our future interactions and cooperative partnerships are often based, at least partially, on observing static images only. Examples include, but are not limited to, the evaluation of job candidates or potential romantic matches on dating apps. In these examples, evaluations of static images are rarely final as they are often followed by a further round of evaluation. However, preliminary screenings based on static photos are frequently used to select potential cooperation partners. This raises the question whether the use of static images for this purpose is a profitable strategy or whether, instead, this practice invites additional bias in the assessment of potential interaction partners. Since there are only a few existing studies that use static facial images as stimuli^26^, we provide new insights into whether these types of images provide clues about the cooperative intent of strangers. Unlike some studies in the literature, our research involves the use of incentivized economic games. Apart from studying the overall detection rate, we also exploit a rich set of individual characteristics to investigate drivers of heterogeneity in judgments and accuracy related to rater and ratee characteristics. In particular, after having completed an economic game that elicits cooperativeness, the Prisoner’s Dilemma (PD), participants of our study were shown 20 facial images of participants in a previous PD experiment. They were asked to evaluate whether the individuals in the images had chosen the cooperative action in that previous experiment. Participants in this rating task were compensated based on the number of correct guesses they made.

In addition to determining whether static facial images signal cooperative intent, our study also investigates whether a shorter exposure time to an image increases or decreases the accuracy of these predictions. Subjects were randomly assigned to one of two treatments: In the *Time pressure* treatment, they were asked to provide a judgment within 5 seconds, whereas in the *Time delay* treatment, they were asked to take at least 5 seconds before submitting their judgment. This approach expands upon research indicating that assessments of facial characteristics are frequently made intuitively^25,27–29^. Prolonged exposure might replace these intuitive judgments with potentially less reliable indicators, such as biases concerning the gender or ethnicity of the potential cooperation partner. Moreover, everyday evaluations of static images of potential partners are often produced in very little time, e.g., mere seconds in the case of dating apps^30^ and a few minutes in the case of CVs^31^. Just 100 ms of exposure to a face are used to judge a person’s reliability, competence, and aggressiveness, although these quick judgments are not necessarily reliable^27,32^. This rapid evaluation process aligns with the expectation that discerning indicators of cooperative behavior is an intuitive and effortless endeavor^29,33^. The dual-process model of decision-making postulates that decisions are the result of two different processes: intuition and reflection^34–37^. Intuition tends to be relatively automatic, fast, effortless, and emotional, while reflection tends to be a slower, more deliberative, controlled, effortful, and a rational calculation-based process. Both types of processes operate concurrently^38–40^. Generally, deliberative processes wield a greater influence on behavior when individuals possess adequate motivation, awareness, and the opportunity to analyze before acting. Intuitive processes are more automatic and therefore exert a greater influence when these conditions are absent^41–43^.

Numerous observable individual characteristics can shape the way we perceive a potential interaction partner. One of the most salient characteristics observable from static facial images is attractiveness^44–46^. Substantial evidence suggests a tendency to associate attractiveness with other positive qualities^44,47–49^, such as intelligence^50^, academic and work performance^51^, and competence^44^. In laboratory experiments, attractive people are often perceived as more cooperative^52,53^ and receive more money in the dictator game^54^. Some of the factors that contribute to the perception of a person as attractive can be assessed more or less objectively^45,55,56^. Two such factors discernible in a photo are facial symmetry^56–58^ and youthfulness^59,60^. However, cooperation in the PD is less prevalent among individuals with more symmetrical facial features^61^. The degree of facial dimorphism, that is, the extent to which a person’s face exhibits more distinctive features of their own sex as opposed to the opposite sex, has frequently been associated with beauty^58,62–64^. In the case of men, a higher degree of masculinity has also been frequently associated with dominance and a propensity for engaging in antisocial behaviors^24,65–68^.

To understand whether cooperative intent can be inferred from static images, we first analyze and report whether participants’ average detection rate is better than chance, in the aggregate and by ratees’ gender and choice in the PD. We then turn to comparing detection rates by treatment condition, revealing whether shorter exposure leads to better detection abilities. Finally, we use regression analyses to investigate the influence of rater and ratee characteristics on the likelihood of accurate assessments, as well as the factors contributing to biases in raters’ judgments of cooperation.

This study contributes to the existing literature in several ways. Firstly, it allows each image to be judged individually rather than requiring a forced choice between two options^24^. This more accurately reflects decisions made in natural settings, where cooperative intent is often assessed independently rather than compared between two alternatives. Furthermore, by incorporating two treatments—one requiring judgments to be made under time pressure and another requiring delayed judgments—our study expands on a relatively small body of research that explores the relationship between exposure time, decision-making speed, and the ability to detect cooperative intentions from static facial images. Previous studies have primarily focused on assessing trustworthiness and have yielded inconsistent results about this relationship, relying on a relatively small pool of raters and ratees^29^. Consistent with the idea that a longer exposure time invites more inferences from irrelevant facial characteristics, accuracy improves with shorter exposure in these studies. Finally, our study also goes beyond the existing literature by considering both the characteristics of raters and the ratees—separately and jointly— when making predictions, thus expanding the understanding of the factors influencing the evaluation of potential cooperation partners.

## Materials and methods

### Participants

The experiment was administered online via Qualtrics, recruiting participants from the Spanish pool of Prolific users. The participant pool was restricted to individuals who have indicated Spanish as their first language and nationality, and with a Prolific rating exceeding 95%. Attention checks were integrated into the study to ensure participant engagement. A total of 300 participants (48% male and 52% female), aged between 18 and 73 years (mean ± SD: $31.53 \pm 10.22$ years), participated in the experiment. The majority of our sample (291) self-identified as Caucasian or Hispanic, with the remainder identifying as Asian (4), Black (1), Arabic (1), Mixed (2) or Other (1). Table 1 summarizes all key demographic variables by treatment condition. It also shows that random assignment worked as expected with little variation in observables between the *Time pressure* and *Time delay* conditions.

**Table 1:** Demographics by treatment group.

	Time pressure	Time delay	Total
Proportion of males	0.479 (0.501)	0.481 (0.501)	0.480 (0.500)
Proportion of Non-Spanish	0.014 (0.119)	0.025 (0.157)	0.020 (0.140)
Age	32.571 (11.399)	30.613 (8.992)	31.527 (10.216)
CRT-Score (0–3)	1.221 (1.004)	1.181 (0.896)	1.200 (0.947)
Risk Tolerance (0–10)	5.107 (2.125)	4.944 (2.241)	5.020 (2.185)
Observations	140	160	300

Rater characteristics: sample means and standard deviations.

The study lasted roughly 20 min. Subjects earned $\pounds$2.80 on average, including a $\pounds$1.70 participation fee. Informed consent was obtained from all participants before the experiment. The ethics committee of the University of Birmingham approved the experimental procedures. All methods were performed in accordance with the relevant guidelines and regulations.

### Experimental design

The experiment consisted of three parts, with the first two parts forming the core of this paper. Part 1 included a simultaneous and anonymous Prisoner’s Dilemma (PD) game. This work-horse two-player game offers two options: to cooperate or to defect (called ’invest’ and ’not-invest’ within the experimental instructions). Mutual cooperation results in a payoff of 90 points for both players. However, there exists an incentive for each player to deviate from mutual cooperation. Unilateral deviations lead to a payoff of 160 points for the deviating player and only 10 points for the cooperating player. As this represents a dominant strategy for each player, the only equilibrium of this game is when both players defect, each earning 30 points. These payoffs were sourced from previous PD experiments^61,69^. The goal of Part 1 was to familiarize participants with the PD framework and to collect data on (1) their cooperative behavior and (2) their beliefs about the cooperative behavior of other participants. Neutral language was used in the task framing, and participants’ comprehension was assessed through a set of quiz questions. Participants could only progress to the decision screen of Part 1 after answering all quiz questions correctly. If this section of the study was randomly selected for payment, participants were matched ex-post, and remunerated based on their choice and the choice of an anonymous partner, with points converted into Euros at a rate of one cent per point.

The primary focus of this study was Part 2, during which we assessed participants’ ability to predict others’ decisions based solely on seeing their static facial photograph. Participants, acting as raters, were shown 20 facial images, randomly chosen from a larger set of 40. These images were those of 40 participants in a previous PD experiment^61^ under the same incentive structure as in Part 1 here. The individuals in the photographs, referred to as ratees for brevity, were all between 20 and 34 years of age (mean ± SD: $24.2 \pm 3.29$ years). Importantly, there was consistency in ethnicity and nationality, with both raters and ratees being recruited from a pool of Spanish nationals and being predominantly Caucasian.

In Part 2, participants were tasked with determining whether ratees had chosen to cooperate or defect when they played the same PD game. We classified ratees as cooperators if they chose the cooperative option, and as defectors if they chose the non-cooperative option in that single PD decision. Detection rates were measured as the proportion of accurate judgments out of the 20 binary decisions presented to each participant (rater). This task was incentivized, offering a reward of 20p for each correct judgment.

The set of static facial images assigned to each rater was balanced in terms of gender (10 males, 10 females) and PD decisions (10 cooperators, 10 defectors) and presented in random order. Additionally, within each gender category, a balance was maintained between cooperators and defectors. We communicated to the raters that there would be an equal proportion of cooperators and defectors in the set of pictures they were evaluating. This was done to avoid participants having to guess the correct cooperation rate in the assessed population in addition to the decision made by each ratee^70^.

Participants were randomly assigned to one of two treatments. In the *Time pressure* condition, participants were instructed to submit their judgment of each image within a 5-s time window, whereas in the *Time delay* condition, they were told to wait at least 5 s before providing their answer. A timer displayed on the screen showed the elapsed time since the participant accessed each rating screen. Time constraints were not strictly enforced, providing participants with the flexibility to deviate in either direction. In the time-pressure literature, there are ongoing debates on whether and how to enforce compliance with time constraints^71,72^. Without any incentives, there is a potential for participants to disregard the manipulation. However, introducing incentives or stringent enforcement can lead to selective compliance or, in the latter case, selection bias due to unrecorded responses. Addressing these issues statistically can be challenging, which led us to opt for not enforcing the time constraints (i.e., an intent-to-treat approach). Our manipulation checks indicate that the majority of participants in our study adhered to the time limits (see the online appendix).

### Measurements

After the main decision task, we elicited several rater’s characteristics summarized in Table 1 including self-declared age and gender. We also assessed raters’ risk tolerance since it may influence both their choices in the PD game of Part 1 and their guessing behavior in Part 2. To that end, we used a self-reported measure of risk attitudes experimentally validated in large samples^73^. Finally, we incorporated participants’ scores on the Cognitive Reflection Test (CRT), hypothesized to be an inverse measure of impulsivity during evaluations^74^. Ratees’ characteristics correspond to those of the 40 participants chosen from a previous PD experiment^61^. These traits encompass age, gender, PD choice, facial dimorphism, self-perceived attractiveness, and Mahalanobis facial asymmetry score. We applied standardized methods to calculate the facial measures from photos of these participants^56,61^. The images were full-face shots taken in a natural position. They were taken with an Olympus E-500 digital camera at a resolution of $4288 \times 2848$ pixels in JPEG format under standardized lighting conditions. The camera was positioned 3 m from the participant and the aperture was fully opened to avoid distortion. Participants were asked to remove any facial adornments and were carefully instructed to look directly into the camera with a neutral expression.

When selecting the 40 photographs for our study, careful consideration was given to ensure a balanced sample of cooperators and non-cooperators with an equal gender distribution across both groups and no differences in the characteristics of interest, as shown in the descriptive statistics in Table SI-1 (Supplementary Information). Correlation coefficients between their facial characteristics are within reasonable bounds ($-0.162$ between attractiveness and facial asymmetry; $-0.199$ between attractiveness and dimorphism and 0.347 between facial asymmetry and dimorphism). Explicit consent was obtained from all individuals whose photographs were used in this study.

### Statistical analyses

To test whether detection rates were better than chance, e.g. 50% of correct judgments, we used one-sided t-tests. When comparing accuracy rates across treatments, we used two-sided Rank-sum tests. For multivariate analyses, we employed panel logit regression models with random effects to study the determinants of participants’ accuracy and biases. These models allow us to find patterns in how images were rated across treatments while accounting for the heterogeneity of raters and ratees and the fact that the former were exposed to multiple stimuli. All statistical tests were performed with Stata v16.0.

## Results

### Detection rates

In Part 1, 56% of participants chose to cooperate despite the one-shot nature of the task. This cooperation rate is in line with those observed in previous PD studies in similar populations^61,69^. Similarly, 66% of participants believed that their interaction partner had cooperated. In line with earlier findings in PD experiments, beliefs and cooperative actions were significantly correlated (pairwise correlation coefficient $\rho =0.61$, p < 0.001).

The main interest of the paper lies in the rating task in Part 2. We first examine whether participants’ detection rate was better than chance. Table 2 provides an answer to this question, summarizing the average share of accurate judgments out of 20.

**Table 2:** Detection rates by treatment condition and ratees’ characteristics.

Ratees	(1) Overall (%)	(2) Time pressure (%)	(3) Time delay (%)
All (#20)	50.8	51.8	49.9
Female (#10)	50.4	49.4	51.3
Male (#10)	51.1	54.1	48.5
Cooperators (#10)	53.7	54.3	53.1
Defectors (#10)	47.9	49.2	46.7

This table provides the percentage of accurate judgments, broken down by ratee characteristics. Row 1 displays the average detection rates for all 20 ratee photographs shown to participants. Rows 2 to 5 contain results grouped by ratee gender (Rows 2 and 3: ten photographs each) and cooperative type (Rows 4 and 5: ten photographs each).

Column 1 combines data from both treatment conditions. On average, participants’ ability to detect choices from facial clues was not statistically better than chance (M = 10.15, SD = 2.17, Cohen’s-d = 0.069, 95% CI [9.91, 10.40], $t_{299}$ = 1.22, p = 0.111, one-tailed). Given the nature of the task, some participants might have perceived the displayed pictures as lacking informative cues about the cooperativeness of the depicted individuals. Alternatively, they might not have paid sufficient attention to the instructions, disregarding the equal presentation of cooperative and non-cooperative faces. In such cases, participants might consistently choose one action (cooperate or defect) in all 20 trials or at least exhibit a significant bias towards one option. Analyzing data from 248 participants who did not favour one of the two options more than 12 times, we find tentative evidence that participants slightly outperformed chance in predicting the actions of the depicted persons (M = 10.22, SD = 2.23, Cohen’s-d = 0.098, 95% CI [9.94, 10.50], $t_{247}$ = 1.54, p = 0.063, one-tailed). Similarly, when excluding data from the 14 participants who selected a specific action (cooperate or defect) more than 17 times out of the 20 trials, the remaining participants still showed a slight advantage over random chance (M = 10.19, SD = 2.20, Cohen’s-d = 0.086, 95% CI [9.93, 10.44], $t_{285}$ = 1.45, p = 0.074, one-tailed).

The subsequent rows of column 1 provide a more detailed analysis of the results, segmented according to the actions or gender of the individuals portrayed in the images (ratee). The gender of the ratee did not significantly influence detection rates for either female (M = 5.04, SD = 1.52, Cohen’s-d = 0.029, 95% CI [4.87, 5.22], $t_{299}$= 0.49, p = 0.622, one-tailed) or male ratees (M = 5.11, SD = 1.62, Cohen’s-d = 0.068, 95% CI [4.93, 5.29], $t_{299}$ = 1.17, p = 0.242, one-tailed). The last two rows offer further analysis of detection rates for cooperative and non-cooperative ratees separately. The detection rate for cooperative ratees significantly exceeded chance (M = 5.37, SD = 1.68, Cohen’s-d = 0.219, 95% CI [5.18, 5.56], $t_{299}$ = 3.79, p < 0.001, one-tailed), while the detection rate for non-cooperative ratees did not exceed chance. Rather, it was significantly below chance (M = 4.79, SD = 1.68, Cohen’s-d = $-0.127$, 95% CI [4.60, 4.98], $t_{299} = -2.20$, p = 0.028, one-tailed).

Next, we ask whether our primary treatment variation had an impact on detection rates. Columns 2 and 3 of Table 2 break down our results by treatment and ratee characteristics. Overall results in row 1 indicate that detection rates were better than chance under time pressure (M = 10.35, SD = 2.30, Cohen’s-d = 0.152, 95% CI [9.97, 10.73], $t_{139}$ = 1.80, p = 0.037, one-tailed). In contrast, under *Time delay*, detection rates did not show a significant improvement over chance ((M = 9.98, SD = 2.05, Cohen’s-d = $-0.009$, 95% CI [9.66, 10.30], $t_{159} = -0.12$, p = 0.546, one-tailed). When we break down the results by ratee characteristics, we observe that participants performing under time pressure displayed an ability to correctly detect the actions of male ratees (M = 5.41, SD = 1.62, Cohen’s-d = 0.251, 95% CI [5.14, 5.68], $t_{139} = 2.97$, p = 0.0018, one-tailed) and cooperative ratees (M = 5.43, SD = 1.70, Cohen’s-d = 0.253, 95% CI [5.15, 5.71], $t_{139}$ = 2.99, p = 0.0017, one-tailed) that was better than chance. The same was not true for female and non-cooperative ratees.

Under *Time delay*, participants still performed better than chance in detecting the actions of cooperative ratees (M = 5.31, SD = 1.66, Cohen’s-d = 0.188, 95% CI [5.05, 5.57], $t_{159}$ = 2.38, p = 0.0092, one-tailed), but did not perform better than chance for non-cooperative ones. The gender of the ratees had no impact on detection abilities under *Time delay*.

Finally, comparing detection rates across columns 2 and 3 reveals that participants in the *Time pressure* condition sometimes exhibited higher detection rates than those in the *Time delay* condition. This difference was not statistically significant for the overall sample (Rank-sum test, z = 1.346, p = 0.178, two-tailed), even after excluding the 14 participants who chose one of the two options 17 times or more (Rank-sum test, z = 1.389, p = 0.165, two-tailed). But breaking down the analysis by the gender of the ratees shows that participants in the time pressure condition had significantly better detection rates for male faces (All: Rank-sum test, z= 2.603, p = 0.0092; Systematic: Rank-sum test, z = 2.631, p-value = 0.0085, two-tailed), but not for female faces (All: Rank-sum test, z = $-1.003$, p = 0.316; Systematic: Rank-sum test, z = $-0.977$ p = 0.329, two-tailed). There were no significant treatment differences when separately analyzing cooperative and non-cooperative ratees.

We note in passing that the small differences between the *Time pressure* and *Time delay* condition are not due to a weak manipulation (see Fig. SI-1 in the SI). On average, response times (per rating) were significantly faster (3.36 s) in the former compared to the latter (6.91 s), although compliance with the time manipulation was not perfect (Rank-sum test, z = $-11.274$, p < 0.001, two-tailed). In Table SI-2 of the SI, we provide further robustness checks showing that the average treatment effect on the treated (ATT), that is, the effect for those affected by the treatment, follows similar patterns as the intent-to-treat (ITT) effect: we find weak evidence that slowing down decisions reduced choice accuracy, which was mostly driven by incorrect judgments about male ratees and non-cooperative ratees (see also Fig. SI-2).

### Rater characteristics and accurate judgments

**Fig. 1:**
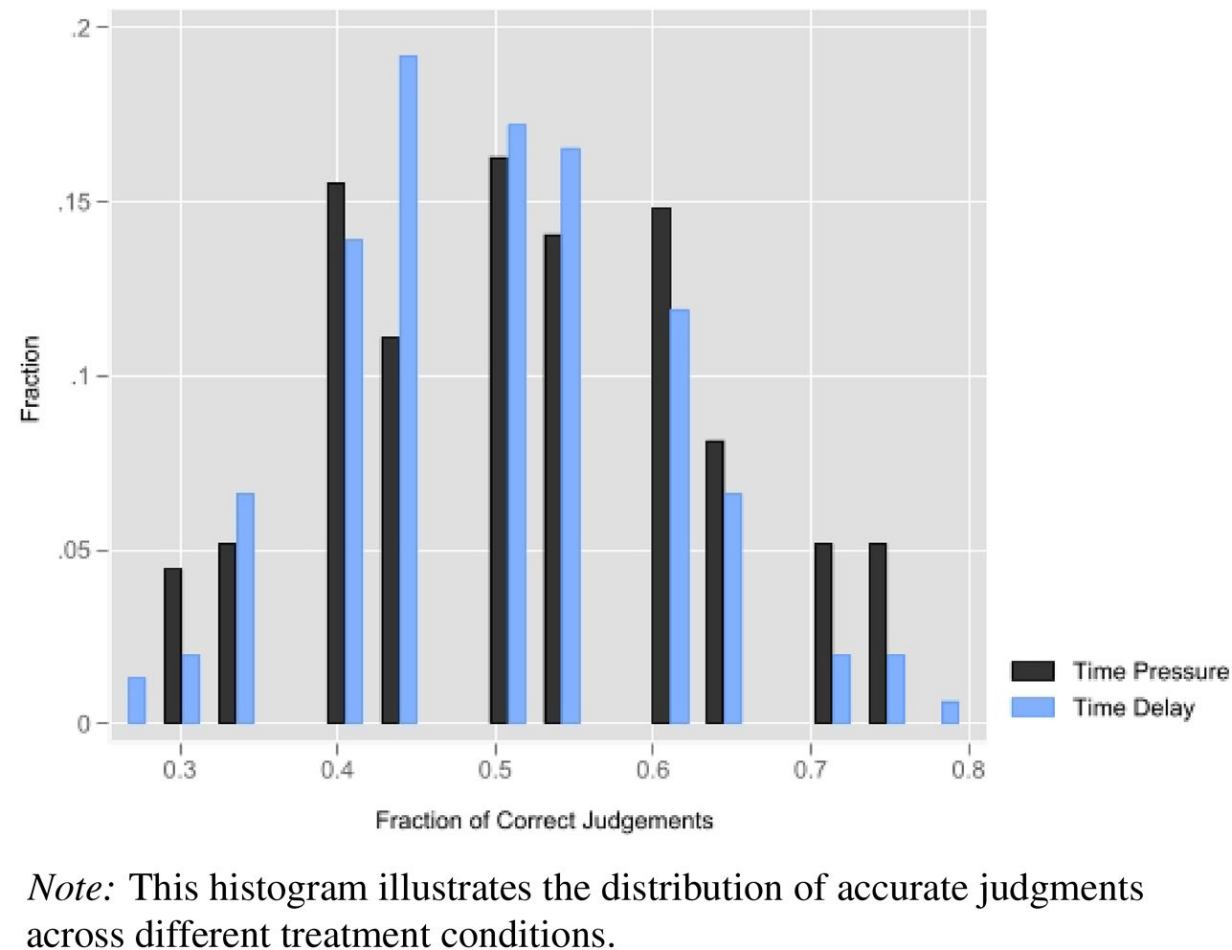
Histogram of Accurate Judgments By Treatment.

We observe significant heterogeneity in raters’ ability to correctly detect cooperators. Figure 1 summarizes the distribution of accurate judgments separately for the *Time pressure* and *Time delay* conditions.

The highest-performing raters accurately evaluate 80% of ratee’s actions (16/20), in contrast to the lowest-performing raters, who only make correct judgments 25% of the time (5/20). As observed previously, the introduction of a time delay shifts this distribution slightly leftward, resulting in a greater proportion of participants scoring below the random-chance benchmark of 50% accuracy (10 out of 20).

In this section, we explore whether and which rater characteristics can account for this heterogeneity. Table 3 contains the results of a panel logit regression, with correct judgments as the dependent variable. The table reports odds ratios for the treatment indicator and various rater characteristics.

**Table 3:** Accuracy: rater characteristics.

	(1) Overall	(2) Female	(3) Male	(4) Defector	(5) Cooperator
Treatment (1 = Delay)	− 0.0771 (− 1.52)	0.0778 (1.08)	− 0.233 (− 3.16)***	− 0.132 (− 1.72)*	− 0.0244 (− 0.31)
Male (1 = Yes)	0.0781 (1.59)	− 0.0254 (− 0.36)	0.183 (2.50)**	0.0988 (1.30)	0.0596 (0.77)
Cooperator (1 = Yes)	− 0.00193 (− 0.03)	0.0375 (0.42)	− 0.0416 (− 0.43)	− 0.0321 (− 0.36)	0.0282 (0.29)
Cooperative belief (1 = Yes)	− 0.0411 (− 0.65)	− 0.0761 (− 0.76)	− 0.00610 (− 0.06)	− 0.304 (− 3.11)***	0.222 (2.14)**
Age (Standardized)	0.0178 (0.75)	0.00837 (0.24)	0.0274 (0.73)	0.00453 (0.12)	0.0317 (0.93)
CRT (> Median)	− 0.00385 (− 0.07)	0.0286 (0.40)	− 0.0365 (− 0.48)	− 0.0584 (− 0.72)	0.0508 (0.63)
Risk Tolerance (> Median)	− 0.0722 (− 1.44)	− 0.0420 (− 0.60)	− 0.103 (− 1.39)	− 0.0480 (− 0.62)	− 0.0987 (− 1.27)
Constant	0.123 (1.60)	− 0.00521 (− 0.05)	0.253 (2.48)**	0.257 (2.54)**	− 0.00761 (− 0.07)
Order Dummy	YES	YES	YES	YES	YES
Observations	6000	3000	3000	3000	3000
Prob $> \chi ^2$	0.192	0.922	0.001	0.002	0.123

Dependent variable: Correct judgment (1 for correct, 0 for incorrect).

This table presents odds ratios from a logit regression with s.e. clustered at the individual level.

*t* statistics in parentheses are used to determine the statistical significance of the respective odds ratios.

Significance levels: *$$p<0.10$$, **$$p<0.05$$, ***$$p<0.01$$, ****$$p<0.001.$$

Considering the full model in column 1 first, neither the treatment nor any of the included rater characteristics influence the likelihood of a correct judgment. Significant associations arise in the remaining columns that split the sample according to ratees’ attributes (gender and cooperative type). Columns 2 and 3 show that male participants exhibited a significant advantage over female participants in detecting the cooperative type of male ratees, while the same was not true for the ability of female participants in detecting the behavior of female ratees. Moreover, the odds of making an accurate judgment about male ratees were reduced under time delay relative to time pressure.

Columns 4 and 5 show an association between beliefs in Part 1 of the experiment and detection ability in Part 2. Specifically, those participants who believed that their partner in the PD of Part 1 had cooperated displayed a worse ability to correctly identify defectors and a better ability to correctly identify cooperators. Other individual characteristics such as CRT-score, age, and risk tolerance did not influence the odds of making an accurate judgment.

### Ratee characteristics and correct judgments

**Fig. 2:**
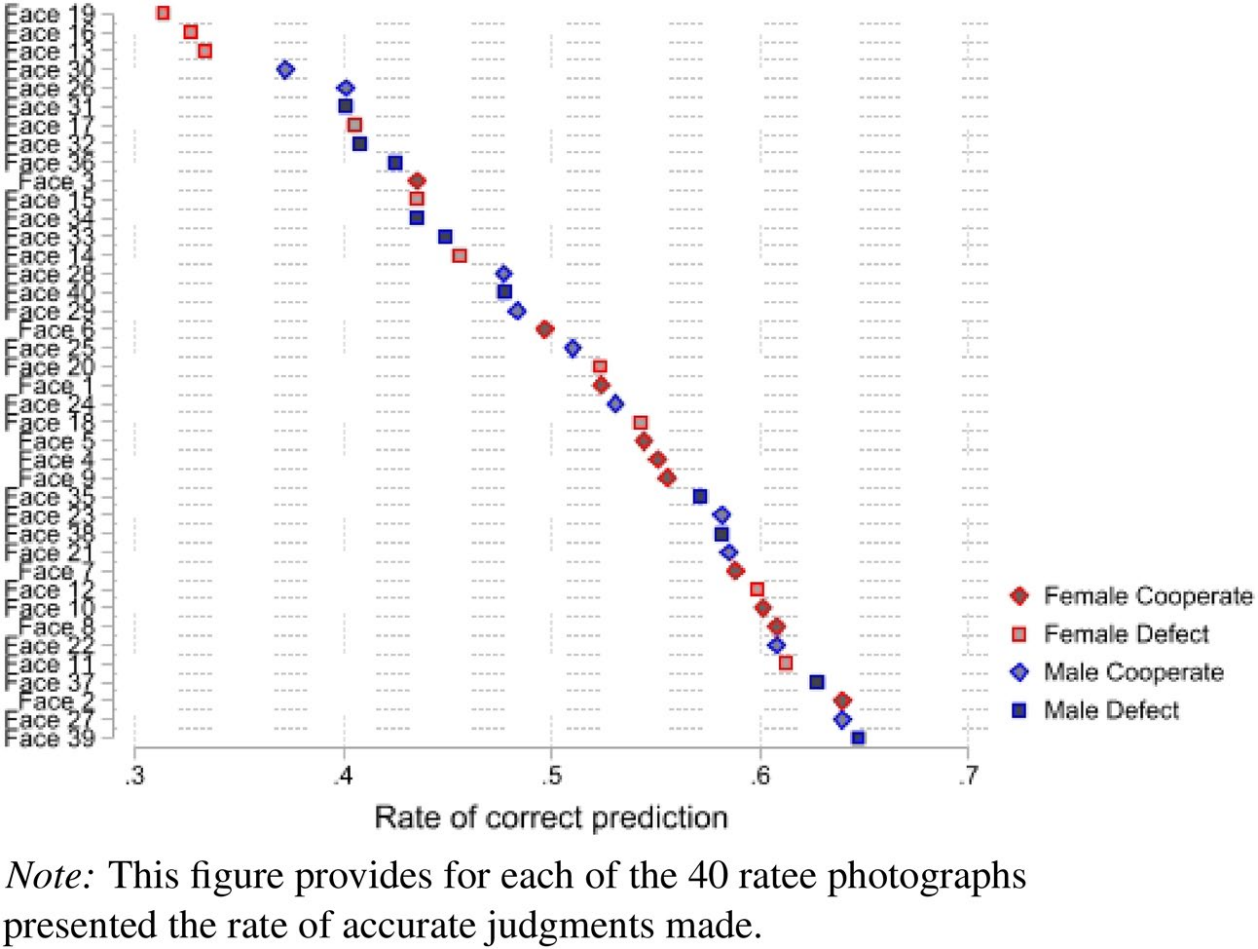
Accurate judgment rate by image.

Raters could base their judgments on some characteristics (e.g., age or gender) inferred from the ratees’ photographs. Figure 2 below shows that there was significant heterogeneity in detection rates across images. This suggests that, rather than random guessing, which would lead to detection rates clustering around 50% for each image, the raters systematically relied on certain characteristics when predicting the actions of the ratees. For example, Figure 2 indicates that female defectors were relatively overrepresented in the lower tail of the distribution of detection rates (below 40% accuracy), while female cooperators were relatively underrepresented. No ratee photograph allowed perfect identification or was perfectly deceptive. This again suggests that cues, if existent, are subtle and not uniformly discernible.

In Table 4, we augment the regression models in Table 3 with a set of observable ratee characteristics. We also control for all unobserved heterogeneity in facial characteristics by including a fixed effect for each image. Results indicate that several ratee characteristics can account for the heterogeneity in detection rates across images. First, male cooperators were harder to correctly identify compared to male defectors, and vice versa. An increase in the ratees’ age significantly worsened the detection rates in all specifications, apart from model 5 (cooperative ratees). In this respect, it is important to point out that the age of ratees only ranged from 20 to 34 years (average 24.2).

**Table 4:** Accuracy: rater and ratee characteristics.

	(1) Overall	(2) Female	(3) Male	(4) Defector	(5) Cooperator
Panel A: rater characteristics					
Treatment (1=Delay)	− 0.0798 (− 1.52)	0.0809 (1.08)	− 0.241 (− 3.16)***	− 0.138 (− 1.72)*	− 0.0251 (− 0.31)
Male (1 = Yes)	0.0809 (1.59)	− 0.0264 (− 0.36)	0.188 (2.51)**	0.104 (1.31)	0.0610 (0.77)
Cooperator (1=Yes)	− 0.00202 (− 0.03)	0.0390 (0.42)	− 0.0430 (− 0.43)	− 0.0336 (− 0.36)	0.0289 (0.29)
Cooperative Belief (1 = Yes)	− 0.0425 (− 0.65)	− 0.0791 (− 0.76)	− 0.00622 (− 0.06)	− 0.318 (− 3.10)***	0.227 (2.14)**
Age (Standardized)	0.0184 (0.75)	0.00868 (0.24)	0.0283 (0.73)	0.00469 (0.12)	0.0325 (0.93)
CRT (> Median)	− 0.00399 (− 0.07)	0.0297 (0.40)	− 0.0377 (− 0.48)	− 0.0617 (− 0.73)	0.0521 (0.63)
Risk tolerance (> Median)	− 0.0747 (− 1.44)	− 0.0436 (− 0.60)	− 0.106 (− 1.39)	− 0.0501 (− 0.62)	− 0.101 (− 1.27)
Panel B: ratee characteristics					
Age (Standardized)	− 0.292 (− 2.61)***	− 0.620 (− 3.60)****	− 0.294 (− 2.61)***	− 0.297 (− 2.61)***	− 0.462 (− 1.45)
Cooperator (1 = Yes)	0.422 (1.37)	1.586 (3.70)****	− 1.122 (− 2.77)***		
Male picture (1 = Yes)	− 0.720 (− 1.61)			− 0.0503 (− 0.17)	− 0.0110 (− 0.05)
Mahalanobis (standardized)	0.487 (3.67)****	0.375 (2.22)**	0.490 (3.67)****	0.495 (3.66)****	0.495 (1.45)
Dimorphism (standardized)	0.656 (3.28)***	− 0.556 (− 3.52)****	0.661 (3.28)***	0.667 (3.28)***	− 0.205 (− 0.77)
Attractiveness (standardized)	− 0.0780 (− 0.98)	− 0.457 (− 4.56)****	− 0.0786 (− 0.98)	− 0.0794 (− 0.98)	− 0.155 (− 0.30)
Constant	0.449 (0.76)	− 1.872 (− 4.06)****	1.340 (2.95)***	0.449 (1.40)	− 0.241 (− 0.92)
Face FE	YES	YES	YES	YES	YES
Order FE	YES	YES	YES	YES	YES
Observations	6000	3000	3000	3000	3000
Prob $> \chi ^2$	0.000	0.000	0.000	0.000	0.000

Dependent variable: Correct judgment (1 for correct, 0 for incorrect).

This table presents odds ratios from a logit regression with s.e. clustered at the individual level.

*t* statistics in parentheses are used to determine the statistical significance of the respective odds ratios.

Significance levels: *$$p<0.10$$, **$$p<0.05$$, ***$$p<0.01$$, ****$$p<0.001.$$

We also include metrics of facial symmetry, gender dimorphism, and self-assessed attractiveness. We first focus on gender dimorphism, which measures the divergence of the depicted face from the average features of the opposite sex; thus, higher scores denote more masculinity in males and more femininity in females. In single-gender regressions (columns 2 and 3), higher dimorphism implies a greater similarity to the facial attributes of that specific gender. In mixed-gender regressions (the remaining columns), higher scores suggest a stronger resemblance to the facial features typical of the own gender. Here, we observe that a higher resemblance to gender-specific facial features enhanced the overall detection of ratees’ type, more specifically for males and non-cooperative ratee’s. In contrast, a higher degree of dimorphism in females significantly reduced correct identification. In simpler terms, males ratees appearing relatively more masculine were identified correctly more often, whereas females appearing more feminine were less likely to be correctly identified.

We next examine the impact of facial symmetry, measured using the Mahalanobis distance. This metric assesses the symmetry in each individual’s facial features by quantifying how much they deviate from a face with the expected symmetry in the population of reference; higher values indicate lower symmetry. Our findings indicate that ratees with less symmetric faces, as denoted by higher Mahalanobis scores, were identified with higher accuracy. This observation is consistent across both genders. While the effect is similar in direction and strength between cooperative and defective ratees, it only reaches statistical significance for defectors.

Lastly, our analysis reveals that while facial attractiveness does not affect rating accuracy in the full sample of images, there is a pronounced negative effect for female ratees. Specifically, female ratees who perceive themselves as more attractive tend to be identified correctly less often. This effect is not observed among male ratees.

### Identifying biases: the role of rater and ratee characteristics in judgments

This section explores through regression models how the characteristics of raters and ratees affected judgments (rather than judgment accuracy). Given the balanced presentation of cooperative types and the absence of differences in ratees’ characteristics across types, any variable showing significance in these regressions indicates that it generated a systematic bias in raters’ evaluations. As expected if participants followed our instructions closely, 52% of all judgments were in favor of cooperation and 48% in favor of non-cooperation.

Table 5 presents the outcomes of various random effects logit models where the dependent variable takes the value 1 if the rater judged the person in the photograph to be a cooperator. All results reported in the table represent odds-ratios.

**Table 5:** Cooperative judgments: rater and ratee characteristics.

	(1) Overall	(2) Time pressure	(3) Time delay	(4) Female	(5) Male
Panel A: rater characteristics					
Male (1 = Yes)	− 0.0197 (− 0.33)	− 0.0379 (− 0.45)	− 0.00409 (− 0.04)	− 0.0288 (− 0.31)	− 0.0114 (− 0.13)
Cooperator (1 = Yes)	0.0276 (0.39)	0.0884 (1.08)	− 0.0313 (− 0.26)	0.0752 (0.73)	− 0.0181 (− 0.16)
Cooperative belief (1 = Yes)	0.271 (3.34)****	0.248 (2.91)***	0.321 (2.29)**	0.223 (1.93)*	0.334 (2.75)***
Age (standardized)	0.0114 (0.42)	− 0.00198 (− 0.06)	0.0280 (0.51)	0.0448 (1.04)	− 0.0214 (− 0.52)
CRT (> Median)	0.0533 (0.84)	0.0204 (0.23)	0.0945 (0.99)	0.160 (1.69)*	− 0.0489 (− 0.52)
Risk tolerance (> Median)	− 0.0284 (− 0.47)	0.0567 (0.66)	− 0.0979 (− 1.03)	0.0120 (0.13)	− 0.0707 (− 0.79)
Panel B: ratee characteristics					
Age (standardized)	0.297 (2.61)***	0.390 (2.05)**	0.224 (1.60)	0.648 (3.61)****	0.305 (2.61)***
Male (1 = Yes)	0.967 (1.93)*	1.026 (1.32)	0.980 (1.43)		
Mahalanobis (standardized)	− 0.495 (− 3.66)****	− 0.585 (− 2.87)***	− 0.433 (− 2.34)**	− 0.392 (− 2.22)**	− 0.508 (− 3.65)****
Dimorphism (standardized)	− 0.667 (− 3.28)***	− 0.880 (− 2.59)***	− 0.511 (− 1.99)**	0.581 (3.53)****	− 0.685 (− 3.29)***
Attractiveness (standardized)	0.0793 (0.98)	− 0.0150 (− 0.13)	0.158 (1.39)	0.478 (4.57)****	0.0814 (0.98)
Face FE	YES	YES	YES	YES	YES
Order FE	YES	YES	YES	YES	YES
Observations	6000	2800	3200	3000	3000
Prob $> \chi ^2$	0.000	0.000	0.000	0.000	0.000

Dependent variable: Cooperation judgment (1 for cooperator, 0 for defector).

This table presents odds ratios from a logit regression with s.e. clustered at the individual level.

*t* statistics in parentheses are used to determine the statistical significance of the respective odds ratios.

Significance levels: *$$p<0.10$$, **$$p<0.05$$, ***$$p<0.01$$, ****$$p<0.001.$$

Panel A contains the results for rater characteristics. Apart from the cooperative beliefs elicited in Part 1, none of the individual rater characteristics assessed in the post-experimental questionnaire shows a significant association with judgments when looking at all judgments (column 1). In short, participants with cooperative beliefs were more likely to judge ratees as cooperative.

The remaining models segment the data further, distinguishing by treatment condition (columns 2 and 3) and ratee gender (columns 4 and 5). In each of these additional specifications, we again find little evidence that any rater characteristic biased judgments.

However, Panel B shows across all specifications that participants’ judgments were partially based on specific facial characteristics of ratees. Focusing on column 1 first, it is evident that older ratees were judged as more cooperative; furthermore, age mattered more for female than for male ratees, as shown in columns 4 and 5.

Again, we included three measures derived from the facial images used in our study. Facial asymmetry affects judgments across all specifications: more facially asymmetric ratees were consistently less likely to be judged as cooperators. The remaining models show that facial asymmetry has a more pronounced impact on judgments made under *Time pressure* compared to those made after a *Time delay*. This suggests that facial symmetry is used as an intuitive cue of cooperativeness.

Attractiveness does not matter for judgments overall. However, female ratees who perceive themselves as more attractive are judged to be more cooperative compared to female ratees who perceive themselves as less attractive.

Facial dimorphism has an overall negative effect on judgments again with stronger effects under *Time pressure* than under *Time delay*. Breaking this down by gender reveals that females with more feminine features are perceived as more cooperative, while men with more masculine features are perceived as less cooperative.

In Table SI-3 in the Supplementary Information, we further demonstrate that, excluding age, none of the aforementioned facial characteristics correlates with the actual decisions made by the ratees in our sample. This implies that the raters’ reliance on these characteristics, as suggested by Table 5, leads them to misjudge the behavioral type of ratees. Given our earlier observation that participants’ detection ability modestly improved over chance, this result implies the existence of additional, more subtle facial cues contributing to that improvement.

## Discussion

Our findings suggest an ability to discern the cooperative disposition of individuals shown in static images that is only moderately above chance. This capability varied with the time allotted for participants to scrutinize the images and with the characteristics of both raters and ratees. Differences emerged when examining the accuracy for specific subsets of ratees. Detection rates significantly outperformed chance across both treatments for cooperative ratees but fell below chance when the ratee in the picture did not cooperate. This differential effect was more pronounced with extended deliberation. Whilst the ability to detect non-cooperators was slightly better than random under *Time pressure*, having more time was counterproductive. Put differently, non-cooperators’ faces successfully obscured their type, especially when raters had more time for deliberation.

These results prompt two remarks. First, the observation that allocating more time to evaluate a static image diminishes detection ability is in line with some previous findings on the advantages of unconscious versus conscious responses^75,76^, although this body of research remains debated^77,78^. Our study does not aim to make a general statement on the merits of these two response types; we cannot assess how consciously participants focused on their judgments or how aware they were of their evaluation process during the allotted time. Moreover, in our specific context, 5 s could be sufficient for forming conscious judgments, while unconscious judgments about facial features may already occur within milliseconds. Notwithstanding, our time-pressure design incorporates some features that allow for an evaluation of the advantages of unconscious decisions^79,80^, such as stimulus complexity, participant training, and task-independent success criteria. Although not a central focus of our study, we find suggestive evidence that unconscious judgments can yield better outcomes than conscious judgments in a large sample of facial ratings. More research will be needed to confirm this, for example, by directly assessing whether judgments are made shortly after exposure to stimuli, even with extended evaluation times.

A second noteworthy observation from Table 2 pertains to non-cooperators’ faces appearing deceptive, i.e., only marginally harder to accurately predict than the faces of cooperators. This aligns with the expectation that non-cooperators imitate cooperative traits, resulting in an overall low accuracy rate in predicting cooperation^12^. Despite the subtlety of cooperation indicators, their detectability persists^24,61^. The notion that cooperative indicators manifest themselves in facial features is not surprising from an evolutionary perspective. It is plausible that signs indicating a propensity to cooperate eventually evolve into signals, understood as “any act or structure that alters the behavior of other organisms, evolved due to that effect, and is effective because the receiver’s response has also evolved”^81^. A key evolutionary mechanism to ensure signal honesty is that signals must be costly, allowing only the most capable individuals to bear the cost^82,83^. For example, acts of helping are most often costly^84,85^. Hence, according to costly signaling theory, signals indicating a propensity to cooperate must also signify individual quality^86^, for instance in the context of partner selection^87^. Fixed facial features can indeed be employed to predict behavior; for example, the detection of trustworthy counterparts in the trust game is notably accurate when facial images lack chromatic information^33,88,89^. When judgments of trustworthiness include information beyond a facial image, detection ability diminishes, as raters then resort to explicit judgments about the perceived reliability of the person in the photograph.

We also observe that the behavior of male ratees is detected more accurately under limited exposure time. This result partially aligns with those obtained when studying the impact of rater characteristics on detection ability. The significant heterogeneity in the ability to detect cooperation has been associated with several rater traits^9,17–20,61^. For instance, the sex of the evaluator influences their ability to detect cooperators and defectors in images taken at the time of the decision^22^, and their judgments of trustworthiness^89^. Table 3 confirms men’s higher precision in assessing the behavior of other men, whereas men and women are not different in their ability to evaluate the cooperative type of women. This aligns with previous findings highlighting the impact of ratees’ characteristics, such as gender or facial dimorphism^24^. Although there are no gender differences in cooperation rates^90^, meta-analyses show that men exhibit a higher level of intragroup cooperation^91,92^. This enhanced intragroup cooperation is commonly associated with ancestral pressures like hunting and intergroup conflict^93^. Selective pressures, which persisted until recently in an evolutionary time-frame, might thus explain men’s enhanced capacity to detect the cooperative intent of male counterparts.

Regarding raters’ characteristics, our results are in line with those observed in the aggregate in previous studies. Increasing deliberation time diminishes predictive ability, particularly for evaluating men and non-cooperators. A crucial finding is the significant impact of participants’ beliefs about their opponent’s behavior in the PD. Those participants who expected their counterpart to cooperate in the PD of Part 1 displayed an enhanced detection ability for cooperative ratees, while those who expected their counterpart to defect in Part 1 were better at spotting non-cooperators among the pool of ratees. In essence, beliefs regarding the behavior of an unknown individual in an anonymous PD game correlate with improved detection of that behavior. Cooperators have a strong interest in detecting fellow cooperators. But for defectors, detecting other non-cooperators should be less important, as they typically prioritize self-interest. However, our findings suggest that non-cooperators develop an ability to detect fellow defectors, possibly because these are less likely to retaliate in the future. In other words, in addition to the well-known presence of conditional cooperators^94^, our results suggest that there could be conditional non-cooperators, who cooperate to avoid exposure for not following a social norm of cooperation. It is crucial to highlight that the correlation we identify between beliefs and detection capability is not attributable to the former skewing guesses in the detection task. Such behavior cannot improve detection rates given that half of the images presented cooperators. For example, if a participant anticipated that 60% of potential partners would cooperate and predicted that 60% (or any percentage greater than 50%) of the images viewed were cooperators, their overall detection rate would amount to $(60\% \times 50\%)+(40\% \times 50\%)=50\%$.

With respect to the characteristics of ratees, several noteworthy observations emerge. First, all relevant raters’ characteristics maintain significance, along with the impact of extended evaluation time. The findings in Table 4 diverge from those presented in Table 2 in that we no longer observe that overall cooperators are more likely to be detected than defectors. This appears to be due to the fact that we are now controlling for facial symmetry and dimorphism, two traits that affected the cooperative choices of the individuals in the photographs^23,61,69,95^ and display a significant impact on detection rates.

Specifically, high dimorphism enhances the detection of non-cooperators and males, while it has the opposite effect in females. These results align with the male warrior hypothesis and the debate on the relationship between dimorphism, beauty, and social status in men^64,91^ and women^96^. Shifting the focus, facial asymmetry improves the detection of female ratees, while age hinders the identification of cooperators. Theoretical explanations for these results pose challenges, hinting at the presence of systematic biases in judgments of cooperation.

Table 5 reveals that several variables are associated with biases in judging a person as a cooperator. Individuals who expected cooperation (defection) from their counterpart in Part 1 were more likely to judge the individuals shown in the photographs as cooperators (defectors). This bias explains the results on detection rates by ratees’ cooperative type in Table 4.

Other biases include perceiving older individuals, particularly older women, and females with more feminine features as more cooperative. This aligns with research suggesting that age and dimorphism are related to beauty^58,62–64,91^. Attractive individuals enjoy advantages in human social interactions^50,97^ and are generally seen as more cooperative^23,52^. Facial asymmetry also consistently biased judgments, with more asymmetrical faces being judged as less cooperative. This finding contrasts with the common observation in the literature that people with more asymmetric facial features cooperate more often in the PD game^69,95^. Hence, if judgments were based on experience alone^23^, participants should have judged facially asymmetric individuals as more cooperative. This phenomenon may be influenced by the stereotype that attractive individuals are inherently good, as symmetry often correlates with perceived attractiveness. While there was no significant correlation between facial symmetry and self-perceived attractiveness of our ratees, it is worth noting that we found that female ratees who perceived themselves as more attractive were judged more often as cooperative. This partially supports previous findings showing that attractive individuals are seen as more cooperative^56^.

It is important to note that these biases were stronger under limited evaluation time; under *Time delay*, all of the described effects were weaker, and insignificant in the case of age. This is remarkable given that judgments are rapidly established^27,32^ and are unlikely to change following deliberation^78^. While biases are present in both quick and deliberate evaluation processes^98^, our findings align with research indicating that decisions made under stress often rely on biased judgments^99^. Although we did not directly measure stress levels in our participants, time pressure likely played a role, particularly considering that reliability judgments are made in a matter of milliseconds.

In sum, our findings underscore the presence of biases that can hinder the accurate prediction of cooperative behavior. The complex interplay between facial characteristics, gender, and time pressure in shaping these judgments highlights the challenge of identifying cooperators based solely on facial cues. Overall, our results demonstrate that preconceived biases based on facial features often impair, rather than enhance, the ability to identify cooperators.

In conclusion, our results highlight the complexity of predicting cooperation based on static images. Although overall detection rates are not better than chance, certain individuals demonstrate enhanced detection ability under specific circumstances. We must acknowledge the inherent noise in investigating accuracy due to various factors. For instance, while there exists a phenotype often associated with cooperative individuals^23,100^, the cooperative phenotype of both raters and ratees in our study was assessed based on choices made in a single economic game. This may introduce some risk of misclassification that, in turn, may pose significant challenges in evaluating the accuracy of raters. Future studies may circumvent this problem by employing larger sample sizes and/or more comprehensive measures of classifying ratees. Nonetheless, even if a cooperative phenotype likely exists, cooperative behavior often depends on the context, rendering such assessments specific to particular situations and inherently probabilistic. Another consideration is that the raters in our task were informed that 50% of the ratees would be cooperators. This approach was deliberately chosen to avoid any bias in identifying detection rates that could arise from mispredicting the baseline rate of cooperation in a population^8^. However, in real-world settings, this additional source of information is rarely available, which, in turn, is likely to affect individuals’ ability to accurately identify cooperative types in the population. We regard these limitations as promising directions for future research. Given the substantial advantages for both individuals and society in correctly identifying cooperators in real-world contexts, exploring the elements that influence this capability remains a crucial topic for further investigation.

## Supplementary Information

Supplementary Information.

## Data Availability

Data for this study are available under: 10.6084/m9.figshare.25610673.
