# Supplementary material for: The role of facial cues in signalling cooperativeness is limited and nuanced
Source: Sci Rep. 2024 Sep 24;14:22009. doi: 10.1038/s41598-024-71685-9 (PMC11422508; doi:10.1038/s41598-024-71685-9)
Supplement: Supplementary file 1 — Supplementary Information. [file 41598_2024_71685_MOESM1_ESM.pdf]

Supplementary Information: The role of facial cues in signalling cooperativeness is limited and nuanced

Johannes Lohse[1,2,*], Santiago Sanchez-Pages[3], and Enrique Turiegano[4]

[1]Leuphana University Lüneburg, Institute for Economics, Lüneburg, Germany
[2]University of Birmingham, Department of Economics, Birmingham, UK
[3]King's College London, Department of Political Economy, London, UK
[4]Universidad Autonoma de Madrid, Department of Biology, Madrid, Spain
[*]johannes.lohse@leuphana.de

ABSTRACT

This document provides additional information for: "The role of facial cues in signalling cooperativeness is limited and nuanced."

Supplementary Information

Ratees' characteristics

The table below displays the average age, Mahalanobis and Dimorphism of the individuals whose photographs were presented to participants in our study (i.e. ratees). All meaningful pairwise comparisons across groups (Female vs. male; Cooperators vs. defectors; Female cooperators vs. Female defectors; Male cooperators vs. male defectors; Female cooperators vs. male cooperators; Female defectors vs. male defectors) yielded no statistically significant differences.

Table SI-1. Average ratees' characteristics by gender and PD choice

Faces	(1) Age	(2) Mahalanobis FA	(3) Dimorphism
Female	23.58	4.122	5.274
Male	24.80	4.101	6.469
Cooperators	23.51	4.221	5.434
Defectors	24.78	4.019	6.259
Female cooperators	22.61	4.278	5.264
Female defectors	24.37	3.995	5.283
Male cooperators	24.35	4.168	5.592
Male defectors	25.18	4.042	7.236

Note: Table presents average values of age, Mahalanobis facial asymmetry (FA), and facial dimorphism across different groups.

Response times and ITT Analysis

Figure SI-1 summarizes the response time distribution for both treatments. Black bars represent the time pressure treatment and blue bars represent the time delay treatment. While there is some overlap the peaks of both distributions are clearly shifted.

We complement the intent-to-treat (ITT) analysis in the main paper with an instrumental variable (IV) regression, which estimates the average treatment effect on the treated (ATT) under the assumption that random treatment assignment is a valid instrument for response times.

Figure SI-1. Response Times by Treatment

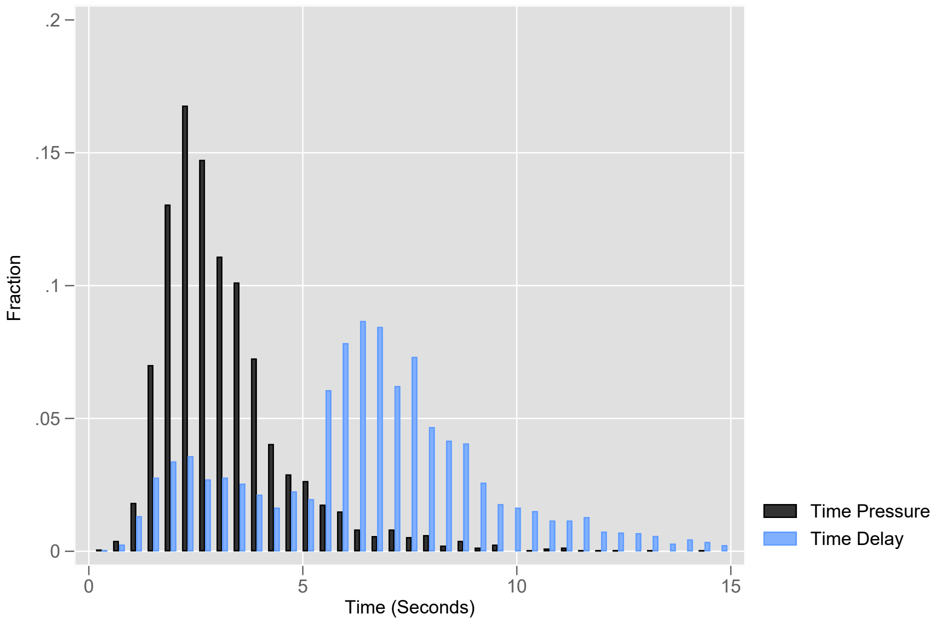

Note: Fraction of responses given at specific times by treatment. We adjusted the x-axis to a maximum of 15 seconds to enhance the clarity of the presentation. This drops roughly 1% of the data which are outliers with response times of up to 325 seconds.

This regression probes whether individuals who make faster decisions due to the treatment exhibit different detection rates compared to those slowed down by the treatment. The subsequent regression table presents results for all images, further delineated by gender and the cooperative type of the person in the picture. We employ a two-stage estimation process. The first stage involves regressing response times against the exogenously assigned treatment variables (refer to Imbens' paper). As anticipated, the time delay condition significantly extends response times. The second stage examines the relationship between response times and accurate judgments. Our findings closely align with those from the ITT analysis. Overall (Column 1), there is no significant relationship between longer response times and accurate judgments. The remaining columns also reveal results consistent with our non-parametric ITT. Participants prompted to spend more time on each judgment are significantly less likely to accurately predict the actions of male faces (Column 3), but this is not the case for female faces (Column 2). Allocating more time to a judgment does not influence the correct identification of defectors (Column 4) or cooperators (Column 5).

The results of this regression are also reflected in the accompanying scatterplots provided below (figure SI-2). The size of each bubble represents the frequency of the respective response times within the response time distribution. The fitted lines illustrate the relationship between response times and ajudgment accuracy. There is a small but discernible negative association for male ratees and defective ratees.

Ratee charateristics and choices

Table SI-3 shows the results of a set of regressions relating facial characteristics of ratees to choices they made in the PD experiment. For the subset of faces displayed in the study, we find no relationship apart from a weak relationship for ratee age among female faces.

Table SI-2. ATT Estimate of the Treatment Effect

	(1) Overall	(2) Female	(3) Male	(4) Defector	(5) Cooperator
Second Stage: Correct Judgment					
Response time	-0.0130	0.0134	-0.0384***	-0.0196	-0.00773
	(-1.45)	(1.07)	(-3.04)	(-1.40)	(-0.59)
Constant	0.0877*	-0.0585	0.230***	0.0515	0.132*
	(1.70)	(-0.86)	(3.21)	(0.65)	(1.81)
First: Response time					
Time delay (1=Yes)	3.555****	3.540****	3.571****	3.362****	3.748****
	(11.80)	(12.86)	(10.06)	(7.89)	(15.00)
Version	0.0158	0.0147	0.121	0.209	-0.157
	(0.05)	(0.05)	(0.30)	(0.48)	(-0.60)
Constant	3.350****	3.299****	3.350****	3.384****	3.306****
	(18.74)	(19.50)	(18.70)	(14.54)	(20.73)
Observations	6000	3000	3000	3000	3000
Prob > χ^2	0.148	0.283	0.002	0.163	0.555

Dependent variable: Correct judgment (1 for correct, 0 for incorrect).

This table presents from an IV Probit regression with s.e. clustered at the individual level.

t statistics in parentheses are used to determine the statistical significance of the respective odds ratios.

Significance levels: * $p < 0.10$, ** $p < 0.05$, *** $p < 0.01$, **** $p < 0.001$

**** $p < 0.001$

Figure SI-2. Response times and Judgment Accuracy

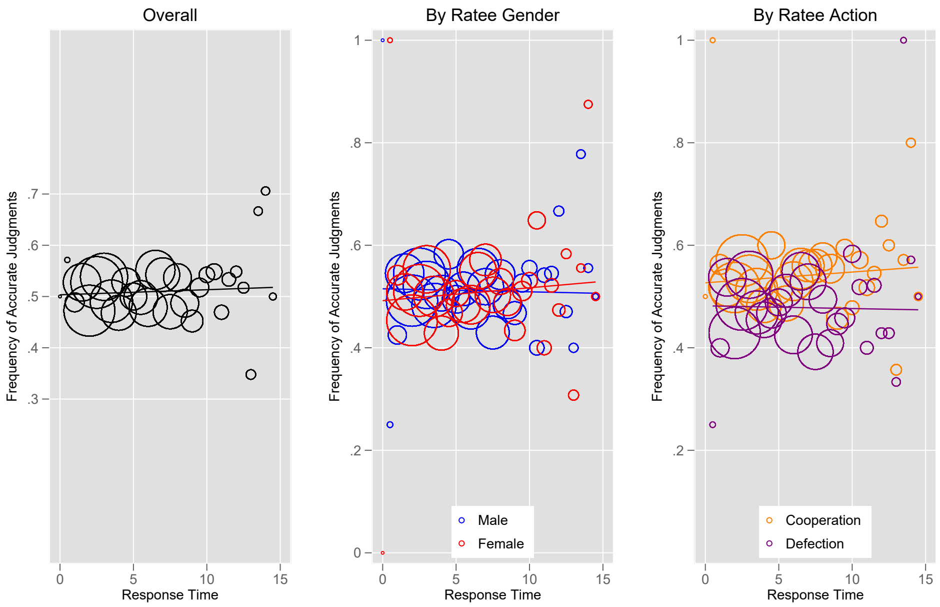

Note: Scatterplots indicating the reltionship between response times and judgment accuracy.

Table SI-3. Ratee Characteristics and Ratee Choices

	(1) Overall	(2) Female	(3) Male
Age	-0.107	-0.217*	-0.000191
	(-1.45)	(-1.87)	(-0.00)
Mahalanobis	0.725	0.611	0.945
	(1.53)	(0.84)	(1.30)
Dimorfism	-0.102	-0.0478	-0.242*
	(-1.18)	(-0.37)	(-1.69)
Attractiveness	-0.0988	0.000514	-0.162
	(-0.81)	(0.00)	(-0.91)
Malepicture	0.168		
	(0.39)		
Constant	0.753	2.829	-1.368
	(0.31)	(0.96)	(-0.34)
Observations	40	20	20

t statistics in parentheses

* $p < 0.10$, ** $p < 0.05$, *** $p < 0.01$, **** $p < 0.001$
